# Peer Review of “Assessment of SARC-F Sensitivity for Probable Sarcopenia Among Community-Dwelling Older Adults: Cross-Sectional Questionnaire Study”

**DOI:** 10.2196/78552

**Published:** 2025-07-25

**Authors:** Anonymous

**Keywords:** sarcopenia, neuromuscular, screening, community, scale, measure, questionnaires, diagnosis, gerontology, older adults, muscular

*This is the peer-review report for “Assessment of SARC-F Sensitivity for Probable Sarcopenia Among Community-Dwelling Older Adults: Cross-Sectional Questionnaire Study.”*

## Round 1 Review

### General Comments

The authors [1] present an intriguing and clinically valuable finding through their receiver operating characteristic (ROC) curve analysis, suggesting that a SARC-F (strength, assistance with walking, rising from a chair, climbing stairs, and falls) score of ≥2 may serve as a new cutoff value for screening probable sarcopenia. This conclusion has significant potential for improving clinical practice by enhancing early detection.

However, the study is based on a relatively small sample size of 204 community-dwelling older adults, and it is unclear if the data were collected from a single center. This limitation raises concerns about the generalizability of the findings to a broader population. I believe the authors could strengthen their argument by conducting additional analyses to address these limitations and provide more robust evidence.

### Major Comments

1. Introduction: Add a discussion on current research gaps (eg, sarcopenia screening) and clearly explain how your study addresses these gaps.
2. Methods: Include additional clinical outcomes such as muscle function, sarcopenia-related symptoms, or quality of life, and compare how thresholds of ≥2 and ≥4 perform in relation to these outcomes.
3. Results: Provide more detailed basic characteristics of participants and compare these between thresholds of ≥2 and ≥4, referring to Malmstrom et al [2] for guidance.
4. Discussion: Update the Discussion to integrate insights from the new results, focusing on the implications of the revised threshold for clinical practice and your limitations.

## Round 2 Review

Thank you for your revisions. I understand that due to the lack of relevant data, you were unable to expand your data analysis. I am pleased to see the addition of Tables 3 and 4 for the subgroup analysis; however, these two tables could be combined. Additionally, you may consider placing the ROC curves from Figures 1 and 2 into a single figure. Using software like MedCalc or SPSS to compare the areas under the different ROC curves would add more depth to the Results section.

## Abbreviations

ROC: receiver operating characteristic
SARC-F: strength, assistance with walking, rising from a chair, climbing stairs, and falls
